# Antibiotic prophylaxis for prevention against Lyme disease following tick bite: an updated systematic review and meta-analysis

**DOI:** 10.1186/s12879-021-06837-7

**Published:** 2021-11-08

**Authors:** Guozhong Zhou, Xin Xu, Yu Zhang, Peng Yue, Shiqi Luo, Yuxin Fan, Jingjing Chen, Meixiao Liu, Yan Dong, Bingxue Li, Jing Kong, Shiyuan Wen, Aihua Liu, Fukai Bao

**Affiliations:** 1 The Institute for Tropical Medicine, School of Basic Medical Sciences, Kunming Medical University, Kunming, 650500 China; 2 Yunnan Province Key Laboratory for Tropical Infectious Diseases in Universities, Kunming Medical University, Kunming, 650500 China

**Keywords:** *Borrelia burgdorferi*, Borreliosis, *Ixodes*, Lyme disease, Tick bite

## Abstract

**Background:**

In areas where Lyme disease is endemic, bites from ticks are common, but no vaccine is currently available against Lyme disease for humans. Therefore, the feasibility of using antibiotic prophylaxis to prevent Lyme disease after a tick bite is worth further exploration. Previous meta-analyses lack sufficient power to demonstrate the efficacy of about antibiotic prophylaxis for the prevention of Lyme disease following a tick bite. In this study, we explored more precise evidence and attempted to identify and update optimum treatment strategies.

**Methods:**

We searched PubMed, Embase, and the Cochrane Library for studies until March 23, 2021. We included studies if the enrolled patients were randomly allocated to a treatment or control group within 72 h following a tick bite and had no clinical evidence of Lyme disease at enrolment. The Preferred Reporting Items for Systematic Reviews and Meta-analyses (PRISMA) reporting guidelines were followed for data abstraction. Two authors (GZZ and XX) independently reviewed the abstracts and identified articles for detailed assessment. We used a random-effects model to calculate the pooled results and reported the 95% confidence interval (CI). Study quality was assessed using a modified Jadad scale, and publication bias was assessed using Egger’s test. We calculated the risk ratio (RR) for the rates of unfavorable events in patients who received intervention versus the control group. This study is registered with PROSPERO, number CRD42021245002.

**Results:**

Six studies (3,766 individuals) were included. The pooled rate of unfavorable events in persons receiving treatment and the control group were 0.4% (95%CI: 0.1–1.1%) and 2.2% (95%CI: 1.6–3.0%), respectively. The pooled RR was 0.38 (95%CI: 0.22–0.66). Subgroup analysis revealed that the pooled RR was 0.29 (95%CI: 0.14–0.60) in the single-use 200-mg doxycycline group; 0.28 (95%CI: 0.05–1.67) in a 10-day course group (Amoxicillin, Penicillin or tetracycline); and 0.73 (95%CI: 0.25–2.08) in a topical antibiotic treatment group (Azithromycin).

**Conclusions:**

The available evidence supports the use of antibiotics for the prevention of Lyme disease, and reveals advantages of using single-dose; however, further confirmation is needed.

## Introduction

Lyme disease is the most common tick-borne disease in the northern hemisphere and is caused by the spirochetes *Borrelia burgdorferi* (*B. burgdorferi*) [1]. The United States has an estimated 300,000 cases of Lyme disease annually [2], and 65,500–85,000 cases are reported annually in Europe [3]. Early manifestations of Lyme disease include non-specific signs and symptoms such as fever, headache, and myalgias. Within days or weeks, untreated infection can spread to other parts of the body, causing more serious neurologic conditions (e.g., meningitis, radiculopathy, and facial palsy) or cardiac abnormalities (e.g., carditis with atrioventricular heart block). Over a period of months or years, untreated infection can lead to arthritis, peripheral neuropathy, or encephalopathy [4, 5].

In areas where Lyme disease is endemic, bites from ticks are common. For instance, 25–30% of people from endemic areas in the United States have reported that a member of the household was bitten by a tick in the preceding year [6]. However, no vaccine for humans is yet available against Lyme disease [7, 8]. Therefore, the feasibility of using antibiotic prophylaxis to prevent Lyme disease following a tick bite is worth further investigation. However, in the past three decades, there have been some controversies over antibiotic prophylaxis to prevent Lyme disease following a tick bite [9–13], and the recommendations of guidelines were conflicting [14]. In 2006 [15], guidelines from the Infectious Diseases Society of America (IDSA) stated that a single dose of 200 mg doxycycline may be offered to adult patients. In 2014 [16], guidelines from the International Lyme and Associated Diseases Society (ILADS) recommended prompt prophylaxis with doxycycline 100–200 mg twice daily for a minimum of 20 days for all *Ixodes* tick bites to the persons who carried evidence of tick feeding, regardless of the degree of tick engorgement or the infection rate in the local tick population. In 2019, guidelines from French Scientific Societies stated that initiating an antibiotic therapy is not recommended, irrespective of the patient’s age, duration of tick attachment, and the stage of development of the extracted tick [7]. In 2020, guidelines from IDSA, American Academy of Neurology (AAN), and American College of Rheumatology (ACR) recommended the administration of a single dose of oral doxycycline within 72 h of tick removal and observation in all age groups [17].

In 1996, Warshafsky et al. published a meta-analysis including three studies and found that the efficacy of antibiotic prophylaxis for prevention of Lyme disease was uncertain, as the 95% confidence interval (CI) was wide [18]. In 2010, Warshafsky et al. updated the meta-analysis, which added one new study into the statistical analysis, and reported that antibiotic prophylaxis was effective for prevention of Lyme disease following a tick bite [19]. However, only 1,082 subjects were included and unfavorable events were scarce in the meta-analysis. Moreover, the efficacy of different treatment strategies could not be assessed. To address these inconsistencies, we performed this updated meta-analysis and explored more precise evidence for an in-depth assessment of the efficacy of antibiotic prophylaxis for prevention of Lyme disease, and attempted to identify the preferred treatment strategy.

## Methods

### Search strategy

We adhered to the PRISMA guidelines for conducting the present meta-analysis, and it was registered in PROSPERO (CRD42021245002). We identified eligible studies by searching PubMed, EMBASE, and the Cochrane Library. We collected studies from the earliest available date, i.e., January 1, 1962 to March 23, 2021. As mentioned in Warshafsky et al. study [19], the following keyword combinations were used: “(Lyme or *borreliosis*) and (prophylaxis or prevention),” without language restrictions. To minimize publication bias, we retrieved the reference lists of included studies and manually searched for other relevant studies that met our inclusion criteria.

### Selection criteria

All studies in which the enrolled patients were randomly allocated to a treatment or control group, were enrolled within 72 h following an *Ixodes* tick bite, and had no clinical evidence of Lyme disease at enrolment were included in our analysis. There were no restrictions based on the antibiotics used, age of the enrolled patients, length of patient follow-up, or the observed outcomes. All included studies were assessed independently by two authors (GZZ and XX). Disagreement for a particular assessment was resolved by discussing the issues with the third partner until a consensus was reached.

### Data extraction

The two authors (GZZ and XX) independently reviewed the abstracts and identified articles for detailed assessment. Disagreement for a particular assessment was resolved by discussing the issues until a consensus was reached. The form included a fixed set of fields: title, author, years of publication, country or area, patient demographics, antibiotics used, daily dose of antibiotics, duration of therapy, duration of follow-up, number of patients in the antibiotic-treatment group and control group, and the number of unfavorable events in each study group. As mentioned in Warshafsky et al. study [19], an unfavorable event was defined as the development of erythema migrans at the site of the tick bite or an objective manifestation compatible with early extracutaneous Lyme disease (e.g., seventh cranial nerve palsy) or late Lyme disease (e.g., arthritis) confirmed by seroconversion.

### Quality assessment

We appraised the quality of studies by using a modified Jadad Scale, which included four parts: randomization, with scores ranging from 0 to 2; concealment of allocation,with scores ranging from 0 to 2; double blinding, with scores ranging from 0 to 2; withdrawals and dropouts, with scores ranging from 0 to 1. Studies with a Jadad score between 1 and 3 were considered low quality, while those with a score between 4 and 7 were considered high quality. The quality of all studies was assessed independently by the same two authors (GZZ and XX). As mentioned, any disagreement was resolved by detailed discussion until consensus was achieved.

### Statistical analysis

We calculated the risk ratio (RR) for the rates of unfavorable events in patients who received intervention versus the control group. Results from studies were grouped according to the treatment strategy of antibiotics, and we also conducted a sensitivity analysis to assess the robustness of results. We used Cochran’s Q test and Higgins I^2^ statistic to assess the heterogeneity of the included studies. We used a random-effects model to calculate the pooled results and the 95% CI. A *p-*value < 0.10 or a I^2^ value > 50% suggested significant heterogeneity. Egger’s test was used to detect publication bias, and a *p-*value < 0.10 on Egger’s test was considered indicative of statistically significant publication bias. This meta-analysis was conducted using the “meta” package in R statistical software version 3.4.3 (Schwarzer, 2007; Team, 2017).

## Results

Initially, 4,515 studies were identified and 4,509 were excluded after screening the titles and abstracts, as well as the full texts of all articles according to the inclusion criteria.

**Fig. 1:**
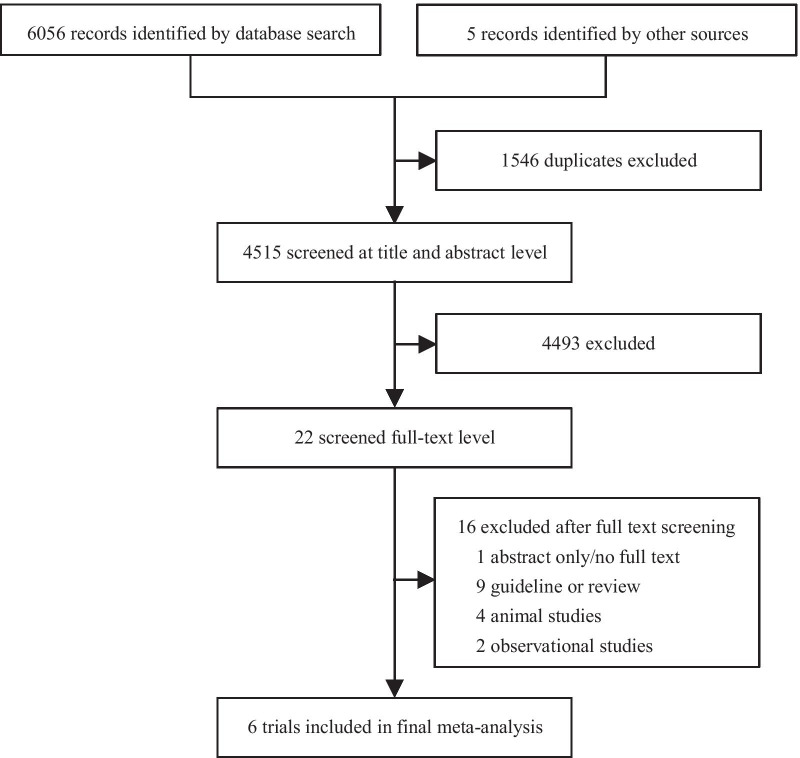
Preferred Reporting Items for Systematic Reviews and Meta-analyses (PRISMA) flowchart of study selection process

Six randomized controlled trials (RCTs) [3, 9, 20–23] met the inclusion and exclusion criteria and were eligible for the final analysis. The selection process is shown in Fig. 1. The characteristics of the included studies are shown in Table 1, and the quality assessment performed by Jadad Scale is shown in Table 2.

**Table 1:** Characteristics of randomized clinical trials included in meta-analysis

	Area	Age	Males (%)	Antibiotic	Oral or topical	Daily dose (mg)	Duration (d)	Follow-up (m)
Costello et al. 1989 [20]	USA	Adults and children	35.7	Penicillin	Oral	1000	10	
Shapiro et al. 1992 [21]	USA	Adults and children	42.6	Amoxicillin	Oral	750	10	12^b^
Agre et al. 1993 [22]	USA	Children	49.2	Penicillin or tetracycline^a^	Oral	1000	10	12–36
Nadelman et al. 2001 [23]	USA	Adults and children	53.3	Doxycycline	Oral	200	1	1.5
Schwameis et al. 2016 [3]	Germany and Austria	Adults	48.7	Azithromyci	Topical	–	3	2
Harms et al. 2021 [9]	Netherlands	Adults and children	50.0	Doxycycline	Oral	200	1	6

^a^Patients older than 9 years received tetracycline and those younger than 9 years received penicillin

^b^Visit follow-up for 3 months, and telephone follow-up for 12 months

**Table 2:** Quality assessment performed by Jadad Scale of included studies

	Costello et al. 1989 [20]	Shapiro et al. 1992 [21]	Agre et al. 1993 [22]	Nadelman et al. 2001 [23]	Schwameis et al. 2016 [3]	Harms et al. 2021 [9]
Randomization	1	2	2	2	2	2
Concealment of allocation	2	2	2	2	2	0^a^
Double blinding	2	2	2	2	2	0^a^
Withdrawals and dropouts	1	1	1	0	1	1
Total Jadad quality score	6	7	7	6	7	3

^a^ Controls did not receive any treatment including placebo, so concealment of allocation and double blinding could not be achieved

**Fig. 2:**
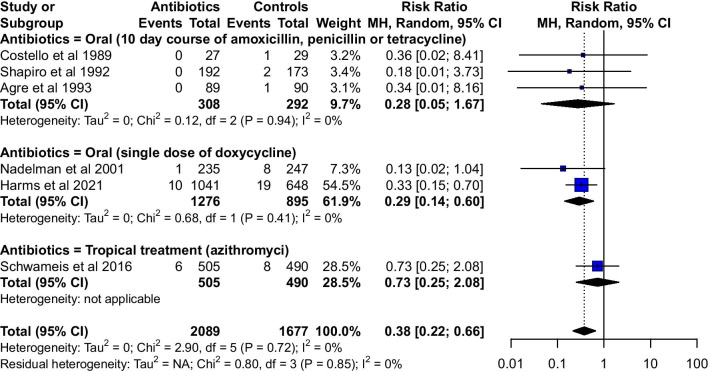
Forest plot of the risk ratio for incidence rates of Lyme disease, with antibiotic groups versus control groups

A total of 3,766 human participants were included in our meta-analysis, and 56 unfavorable events were observed. Of the 56 unfavorable events, 55 were *erythema migrans*, and only one was disseminated Lyme disease. Meta-analysis showed that the pooled rates of unfavorable events in patients who received intervention and control groups were 0.4% (95%CI: 0.1–1.1%, I^2^ = 55%) and 2.2% (95%CI: 1.6–3.0%, I^2^ = 5%), respectively. The pooled RR was 0.38 (95%CI: 0.22–0.66, I^2^ = 0%) (Table 3 and Fig. 2).

**Table 3:** Main outcomes measures, for total calculation and subgroups

	Studies (n)	Participants (n)	Rate in intervention (%)	I^2^ (%)	Participants (n)	Rate in control (%)	I^2^ (%)	Risk ratio	I^2^ (%)
Oral treatment	5	1584	0.2 (0.0–1.0)	57	1187	2.5 (1.7–3.5)	0	0.29 (0.15–0.57)	0
10-day course	3	308	0.0 (0.0–0.3)	0	292	1.3 (0.3–2.9)	0	0.28 (0.05–1.67)	0
Single dose	2	1276	0.8 (0.4–1.4)	0	895	3.0 (2.0–4.2)	0	0.29 (0.14–0.60)	0
Topical treatment	1	505	1.2 (0.4–2.3)	N/A	490	1.6 (0.7–2.9)	N/A	0.73 (0.25–2.08)	N/A
Total	6	2089	0.4 (0.1–1.1)	55	1677	2.2 (1.6–3.0)	5	0.38 (0.22–0.66)	0

Oral antibiotic therapy was administered in five studies, and the pooled rate of unfavorable events in patients who received intervention and control groups were 0.2% (95%CI: 0.0–1.0%, I^2^ = 57%) and 2.5% (95%CI: 1.7–3.5%, I^2^ = 0%), respectively. The pooled RR was 0.29 (95%CI: 0.15–0.57, I^2^ = 0%) (Table 3). Of these five studies, a 10-day course of antibiotic treatment was administered in three studies, and the pooled rate of unfavorable events in patients who received intervention and control groups were 0.0% (95%CI: 0.0–0.3%, I^2^ = 0%) and 1.3% (95%CI: 0.3–2.9%, I^2^ = 0%), respectively. The pooled RR was 0.28 (95%CI: 0.05–1.67, I^2^ = 0%). A single-dose 200-mg doxycycline therapy was administered in the remaining two studies, and the pooled rates of unfavorable events in persons with intervention and control groups were 0.8% (95%CI: 0.4–1.4%, I^2^ = 0%) and 3.0% (95%CI: 2.0–4.2%, I^2^ = 0%), respectively. The pooled RR was 0.29 (95%CI: 0.14–0.60, I^2^ = 0%). Topical antibiotic treatment was administered in only one study, and the pooled rates of unfavorable events in patients who received intervention and control groups were 1.2% (95%CI: 0.4–2.3%) and 1.6% (95%CI: 0.7–2.9%), respectively. The RR was 0.73 (95%CI: 0.25–2.08) (Table 3 and Fig. 2).

**Fig. 3:**
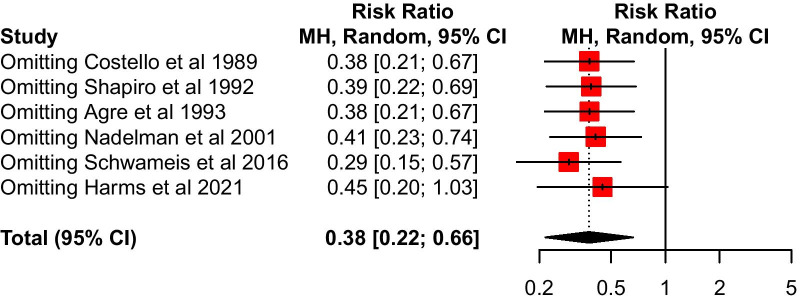
The sensitivity analysis of the six included studies

The results of sensitivity analysis showed that the confidence interval of the pooled RR became imprecise when Harm et al.’s study [9] was excluded (Fig. 3). Egger’s test was used to assess for any publication bias among the six studies, and no evidence of publication bias was found in this review (*p* = 0.515).

## Discussion

Our study included more participants than a previous meta-analysis [19] (3,766 vs. 1,082), and strengthened the evidence that prophylactic antibiotic use is effective for the prevention of Lyme disease following a tick bite. Furthermore, our subgroup analysis revealed that patients who received a single dose (200 mg) course were shown to be less likely to develop Lyme disease than those given placebo (RR, 0.29 [95%CI: 0.14–0.60]), but there is no evidence of the effectiveness of a 10-day course and topical antibiotics course (RR, 0.28 [95%CI: 0.05–1.67] and 0.73 [95%CI: 0.25–2.08]), respectively. Our results support the strategy of a single-dose oral doxycycline therapy for prevention of Lyme disease.

As early as 2001, Nadelman et al. assessed the effect of doxycycline in the prevention of Lyme disease. However, the effectiveness estimated in the RCTs showed a wide confidence interval (RR = 0.13 [0.02–1.04]) [23]. Until recently, an RCT [9] with a relatively large sample size (n = 1,089) provided stronger evidence that a single dose of doxycycline can prevent the development of Lyme disease, following a bite from *Ixodes ricinus* (RR = 0.33 [0.15–0.70]). Our meta-analysis combined two RCTs and showed a more accurate CI (RR = 0.29 (0.14–0.60]). Additionally, two observational studies reported the results of doxycycline in the prevention of Lyme disease. Korenberg et al. [24] reported that none of the patients in the experimental group (n = 261) developed erythema migrans after receiving doxycycline (100 mg twice daily) for 3–5 days after the tick bite, whereas 5/97 patients developed erythema migrans in the control group which did not receive any antibiotics. Jackson et al. [25] reported the clinical application of doxycycline for Lyme disease prophylaxis, and the results indicated a high level of satisfaction with the pharmacy services provided, with no reports of subsequent development of Lyme disease symptoms or other side effects. However, the sample size of this study was small (n = 8).

Although our results support the use of antibiotics for the prevention of Lyme disease and the advantages of a single dose of doxycycline, routine use of antibiotic prophylaxis is not recommended after a recognized tick bite [17]. In our meta-analysis, we estimated that 50 patients (95%CI: 25–100) would need to be treated (NNT) with single-dose doxycycline to prevent one case of Lyme disease. Therefore, it is essential to determine who is at high risk of infection and who is worthy of treatment. For instance, animal studies have shown an exponential increase in the risk of *B. burgdorferi* infection after 48–72 h of deer tick attachment [26, 27]. Consequently, guidelines state that a tick bite is considered to be high-risk only if it was attached for more than 36 h [16]. Falco et al. reported that 52.5% of all tick bites had been attached for < 36 h [28], so the recommendation represents that nearly half of patients avoid receive antibiotics treatment. Additionally, Nadelman et al. [23] found that ticks which were partially engorged with blood (with incidence rate of 9.9%), rather than unfed ticks (incidence rate of 0%), were associated with the development of *erythema migrans*. Nadelman et al. [23] found that *erythema migrans* developed more frequently after bites from nymphal ticks than after bites from adult ticks, with an incidence rate of 5.6% and 0%, respectively. Harms et al. [9] revealed that 11.1% untreated patients with a *B. burgdorferi*-positive tick bite developed Lyme disease, and the NNT in this subgroup was only 10. These findings might provide valuable information for clinicians, but need further confirmation.

Antibiotic use has some side effects [13]. The major side effects of oral doxycycline include enterocolitis, anaphylaxis (including angioedema), Stevens–Johnson syndrome, severe urticarial reactions, and a lupus-like syndrome. Minor reactions of intravenous ceftriaxone include gastrointestinal symptoms of abdominal pain, nausea, vomiting, and diarrhea, and hypersensitivity reactions such as rash, pruritus, fever and chills, candidiasis, and local reactions at the injection site [14]. Although none of included studies reported serious side effects, there were still many minor side effects reported such as rash or nausea. The two studies revealed that incidence of mild side effects after using single-dose doxycycline was 5.9–30.1% [9, 23], which suggests that up to a third of patients are likely to suffer mild side effects. Furthermore, Nadelman et al. found that 18.2% of patients were recognized additional tick bites after enrollment, but during the 6-week study period, the participants needed repeated antibiotic prophylaxis, which would strongly increased the risk of side effects [23]. Availability of a universally acceptable and effective prophylactic agent with minimal side effects would be the ideal. Previous studies found that topical azithromycin was highly effective when applied topically at the sites of tick bites in mice [29, 30]. Although no effective evidences were found in human trials [3], a topical pharmacological prophylactic strategy is still worth exploring [31], given that minor side effects such as localized itching, redness, and dryness were reported only in 1.6% patients [3].

Our meta-analysis has some limitations. First, although we screened more than 4000 related articles, only six studies were eligible for final analysis. Second, we included 4 studies from USA and 2 studies from Europe, the difference of *Ixodes* species and *B. burgdorferi* subspecies between the USA and Europe may bring heterogeneity. Third, *erythema migrans* was considered as the main end point of all included studies evaluating antibiotic prophylaxis, since it is the most common clinical manifestation and only reliable marker of infection caused by *B. burgdorferi* infection. However, this end point was limited and could have resulted in underestimation of the actual incidence of *B. burgdorferi* infection. Fourth, Harm et al. study [9], which included 1689 participants, contributed 54.5% of the weight to the pooled results, but this study was the only one assessed as low quality (Jadad score 3). Therefore, our evidence is limited and further confirmation is needed. Last, we did not analyze the seroconversion results, because only few patients showed seroconversion even in the control group.

## Conclusion

The available evidence supports the prophylactic use of antibiotics for the prevention of Lyme disease and the advantages of a single dose of doxycycline, but further confirmation is needed.

## Data Availability

The datasets used and/or analyzed during the current study are available from the corresponding author on reasonable request.

## Abbreviations

*B. Burgdorferi*: *Borrelia burgdorferi*
CI: Confidence interval
RR: Risk ratio
RCTs: Randomized controlled trials
NNT: Need to be treated
